# Energy landscapes of resting-state brain networks

**DOI:** 10.3389/fninf.2014.00012

**Published:** 2014-02-25

**Authors:** Takamitsu Watanabe, Satoshi Hirose, Hiroyuki Wada, Yoshio Imai, Toru Machida, Ichiro Shirouzu, Seiki Konishi, Yasushi Miyashita, Naoki Masuda

**Affiliations:** ^1^Department of Physiology, The University of Tokyo School of MedicineTokyo, Japan; ^2^Awareness Group, Institute of Cognitive Neuroscience, University College LondonLondon, UK; ^3^Department of Radiology, NTT Medical Center TokyoTokyo, Japan; ^4^Department of Mathematical Informatics, The University of TokyoTokyo, Japan; ^5^CREST, Japan Science and Technology AgencySaitama, Japan

**Keywords:** resting-state network, maximum entropy model, Ising model, attractor dynamics, functional connectivity

## Abstract

During rest, the human brain performs essential functions such as memory maintenance, which are associated with resting-state brain networks (RSNs) including the default-mode network (DMN) and frontoparietal network (FPN). Previous studies based on spiking-neuron network models and their reduced models, as well as those based on imaging data, suggest that resting-state network activity can be captured as attractor dynamics, i.e., dynamics of the brain state toward an attractive state and transitions between different attractors. Here, we analyze the energy landscapes of the RSNs by applying the maximum entropy model, or equivalently the Ising spin model, to human RSN data. We use the previously estimated parameter values to define the energy landscape, and the disconnectivity graph method to estimate the number of local energy minima (equivalent to attractors in attractor dynamics), the basin size, and hierarchical relationships among the different local minima. In both of the DMN and FPN, low-energy local minima tended to have large basins. A majority of the network states belonged to a basin of one of a few local minima. Therefore, a small number of local minima constituted the backbone of each RSN. In the DMN, the energy landscape consisted of two groups of low-energy local minima that are separated by a relatively high energy barrier. Within each group, the activity patterns of the local minima were similar, and different minima were connected by relatively low energy barriers. In the FPN, all dominant local minima were separated by relatively low energy barriers such that they formed a single coarse-grained global minimum. Our results indicate that multistable attractor dynamics may underlie the DMN, but not the FPN, and assist memory maintenance with different memory states.

## Introduction

In the last few decades, a line of neuroimaging studies have accumulated evidence supporting that spontaneous brain activity during rest is not random enough to be averaged out in statistical analysis (Biswal et al., 1995; Raichle et al., 2001; Greicius et al., 2003). The brain activity in resting states shows consistent spatial patterns called the resting-state networks (RSNs) (Raichle et al., 2001; Greicius et al., 2003; Fox et al., 2005; Dosenbach et al., 2006; Fair et al., 2009). Connections between the RSNs and cognitive functions have been revealed in previous studies. In particular, the default-mode network (DMN), one of the representative RSNs, is suggested to be engaged in self-referential mental processes and maintenance of long-term memory (Raichle et al., 2001; Greicius et al., 2003; Buckner et al., 2008; Uddin et al., 2009). The fronto-parietal network (FPN), another RSN, is known to be recruited during cognitive tasks with relatively high loads that require continuous attention (Dosenbach et al., 2006; Corbetta et al., 2008; Fair et al., 2009).

Most of these results on the RSNs were derived from correlations between slow oscillations of brain activity (0.01–0.1 Hz) in different brain regions. However, the neural activity as observed in the RSNs at a macroscopic spatial scale is dynamic on a much shorter time scale. Experimental and computational studies indicate that within a RSN, a group of brain regions is specifically activated within a specific time window, and that different groups of regions are activated during different time windows (Honey et al., 2007; Chang and Glover, 2010; Kiviniemi et al., 2011; Allen et al., 2012; Hutchison et al., 2013). Such spatio-temporal dynamics of the RSNs may facilitate, for example, the flexibility of human cognitive functions (Allen et al., 2012).

These results pertaining to the dynamics of the resting-state brain activity suggest that the activity of the RSNs may be captured in terms of transitions among locally stable states, i.e., attractor states (Deco et al., 2012, 2013; Nakagawa et al., 2013). In fact, beyond the description of RSNs, attractor network models of spiking neurons and firing-rate models derived by the reduction of spiking-neuron models have been used to model cortical dynamics (for reviews, see Barbieri and Brunel, 2008; Wang, 2009; Braun and Mattia, 2010; Knierim and Zhang, 2012). In particular, the role of attractor dynamics has been implicated in brain activity during various cognitive functions such as associative long-term memory, non-spatial working memory, spatial working memory, place field recognition, decision making, and attention. The aforementioned models are particularly successful in describing persistent activity recorded during these cognitive tasks. Although attractor network models may be too simple to describe fast transients of brain activity accurately (Rabinovich et al., 2008; Rabinovich and Varona, 2011), these experimental and numerical results are consistent with the notion that brain dynamics are multistable and that the brain's state travels from one state to another depending on, for example, external input and endogenous cognitive processes.

In associative memory models, an energy function often exists such that each state possesses a corresponding energy value and a state with a low energy is taken with a large probability (Hopfield, 1982; Hertz et al., 1991). In this case, the attractor dynamics can be described by a trajectory that represents a dynamical state of the brain in an energy landscape. Therefore, estimating the energy landscapes of brain activity contributes to understanding of brain dynamics from the perspective of attractor dynamics. In the present study, we investigate the energy landscapes of resting-state brain activity using the functional magnetic resonance imaging (fMRI) data previously collected by our group (Watanabe et al., 2013). In the previous work based on these data, we demonstrated that the so-called pairwise maximum entropy model (MEM) (Schneidman et al., 2006; Shlens et al., 2006; Tang et al., 2008; Yu et al., 2008; Ohiorhenuan et al., 2010; Santos et al., 2010; Ganmor et al., 2011) described the activities of the DMN and FPN with high accuracy (Watanabe et al., 2013). For the fitted models from that study and randomized RSNs, here we calculated the energy of all the brain states and identified local minima of energy that would correspond to the attractors in attractor dynamics. Then, we applied the so-called disconnectivity graph method (Becker and Karplus, 1997) to the empirical and artificial energy landscapes of the RSNs. We found that the energy landscapes of the DMN and FPN are qualitatively different.

## Materials and methods

### Data acquisition and fitting of the pairwise MEM

**Figure 1:**
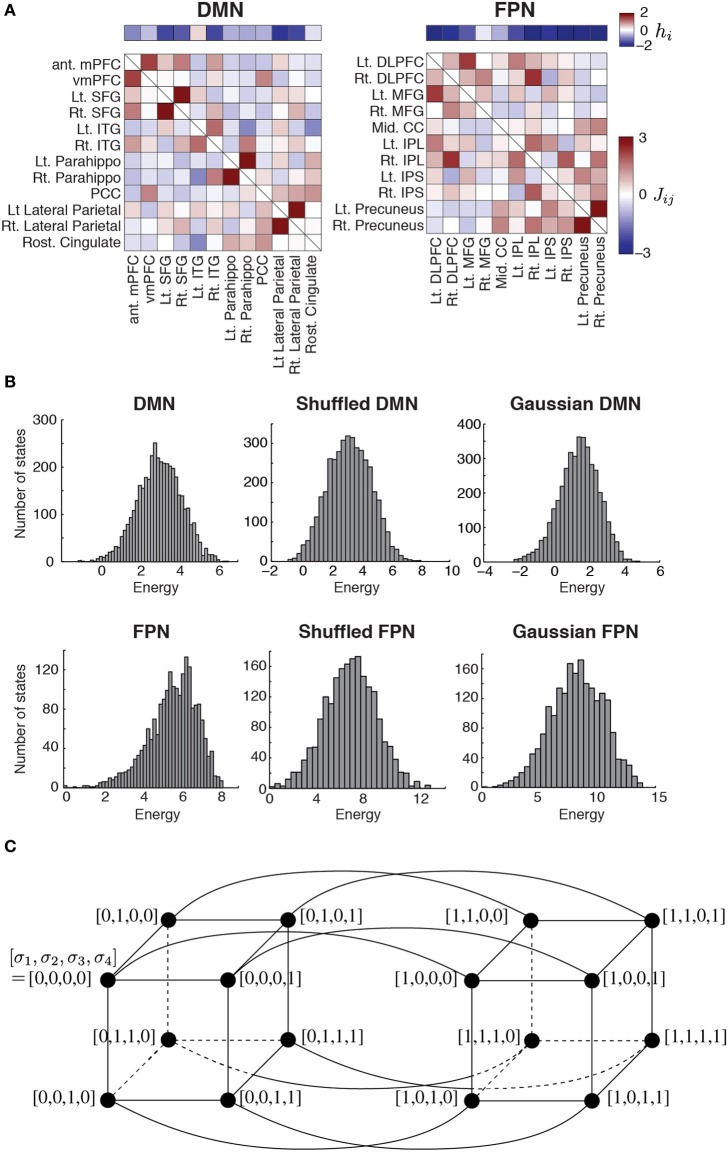
**(A)** Parameter values estimated for the two RSNs. The horizontal bars show the basal brain activity (*h*_*i*_). The square matrices show the functional connectivity between pairs of regions (*J*_*ij*_) as determined by the fitting of the pairwise MEM. The obtained parameter values were identical to those obtained in our previous study (Watanabe et al., 2013). DMN, default mode network; FPN, fronto-parietal network; ant mPFC, anterior medial prefrontal cortex; vmPFC, ventro-medial prefrontal cortex; Lt, left; Rt, right; SFG, superior frontal gyrus; ITG, inferior temporal gyrus; Parahippo, parahippocampal gyrus; PCC, posterior cingulate cortex; DLPFC, dorso-lateral prefrontal cortex; MFG, middle prefrontal cortex; Mid, middle; CC, cingulate cortex; IPL, inferior parietal lobule; IPS, inferior parietal sulcus. **(B)** Distribution of energy for each network. To generate the histograms, we weighted each state equally, i.e., not with the probability that the state is realized. The results for the shuffled and Gaussian networks are based on a single realization of the network. **(C)** Concept of neighbors in a network of network states. For illustration, we set *N* = 4. The circles represent nodes, i.e., network states. A link between a pair of nodes indicates that the two nodes are adjacent.

To examine the energy landscape of the RSNs, we used the parameter values estimated in our previous study in which we fitted the so-called pairwise MEM to the resting-state fMRI data (Watanabe et al., 2013) (Figure 1A). The fMRI data were recorded while six healthy right-handed subjects (aged 20–23 years; three males) were resting inside a 3T MRI scanner (Philips Achieva X 3T Rel. 2.6, Best, The Netherlands; gradient-echo echo-planar sequences: *TR* = 9.045 s, *TE* = 35 ms, flip angle = 90°, resolution = 2 × 2 × 2 mm^3^, 75 slices). In total, 17,820 volumes of resting-state fMRI images were obtained. The entire procedure for the MRI scanning was approved by the institutional review board of The University of Tokyo, School of Medicine.

The pairwise MEM and the fitting procedure are outlined as follows. Readers interested in the detailed procedures should refer to our previous article (Watanabe et al., 2013). First, we conducted a conventional preprocessing procedure that consisted of slice-timing correction, spatial normalization, spatial smoothing, motion correction, and temporal band-pass filtering. Second, to normalize the fMRI data, we subtracted the average from the signals and divided the obtained values by their standard deviation for each brain region. Third, we binarized the normalized signals with a threshold of 0.1. The binarized activity at brain region *i* and discrete time *t*, denoted by σ^*t*^_*i*_, is either active (+1) or inactive (0). The network state at time *t* is described by

(1) $$V^{t}=\left[\sigma_{1}^{t},\sigma_{2}^{t},\cdots ,\sigma_{N}^{t}\right],$$

where *N* is the number of the brain regions in a RSN. It should be noted that there are 2^*N*^ network states. The empirical activation probability of region *i*, denoted by 〈σ_*i*_〉, is equal to $\left(1/T\right)\sum_{t=1}^{T}\sigma_{i}^{t}$, where *T* is the number of images. The empirical joint activation probability of regions *i* and *j*, denoted by 〈σ_*i*_ σ_*j*_〉, is given by $(1/T)\sum_{t=1}^{T}\sigma_{i}^{t}\sigma_{j}^{t}$.

Fourth, we adopted the distribution of the network state that maximized the entropy under the restriction that 〈σ_*i*_〉 and 〈σ_*i*_ σ_*j*_〉 (1 ≤ *i* ≤ *N*, 1 ≤ *j* ≤ *N*, *i* ≠ *j*) for the inferred model were equal to the empirical values. Such a distribution is known to have the form

(2) $$P\left(V_{k}\right)=e^{-E\left(V_{k}\right)}/\sum_{\ell =1}^{2^{N}}e^{-E\left(V_{\ell }\right)},$$

where *P*(*V*_*k*_) is the probability of the *k* th network state *V*_*k*_, and

(3) $$E\left(V_{k}\right)=-\sum_{i=1}^{N}h_{i}\sigma_{i}\left(V_{k}\right)-\frac{1}{2}\sum_{i=1}^{N}\sum_{j=1,j\neq i}^{N}J_{ij}\sigma_{i}\left(V_{k}\right)\text{​}\sigma_{j}\left(V_{k}\right)$$

is the energy of network state *V*_*k*_. Variable σ_*i*_ (*V*_*k*_) indicates the value of σ_*i*_ (i.e., 1 or 0) under network state *V*_*k*_. For the inferred model, the expected activation probability, 〈σ_*i*_〉_*m*_, and the expected pairwise joint activation probability, 〈σ_*i*_ σ_*j*_〉_*m*_, are given by ${\langle \sigma_{i}\rangle }_{\text{m}}=\sum_{\ell =1}^{2^{N}}\sigma_{i}(V_{\ell })P(V_{\ell })$ and ${\langle \sigma_{i}\;\sigma_{j}\rangle }_{\text{m}}=\sum_{\ell =1}^{2^{N}}\sigma_{i}(V_{\ell })\sigma_{j}(V_{\ell })P(V_{\ell })$, respectively. We determined *h*_*i*_ and *J*_*ij*_ by iteratively adjusting 〈σ_*i*_〉_*m*_ and 〈σ_*i*_ σ_*j*_〉_*m*_ toward 〈σ_*i*_〉 and 〈σ_*i*_ σ_*j*_〉, respectively, with a gradient descent algorithm. As a result, we obtained *h*_*i*_(1 ≤ *i* ≤ *N*) and *J*_*ij*_ (= *J*_*ji*_; 1 ≤ *i* ≤ *N*, 1 ≤ *j* ≤ *N*, *i* ≠ *j*) for a RSN (DMN or FPN) (Figure 1A). Here, *h*_*i*_ is considered to represent the basal brain activity of region *i*, i.e., the expected brain activity when the region is isolated. *J*_*ij*_ represents the functional interaction between regions *i* and *j*. The brain regions constituting each RSN, with the labels being indicated in Figure 1A, were determined on the basis of previous studies (Dosenbach et al., 2006; Fair et al., 2009).

### Disconnectivity graph

The energy landscape of a RSN is specified by two factors: the energy *E*(*V*_*k*_) of the 2^*N*^ network states *V*_*k*_, which are regarded as nodes in a network of network states; and the connectivity between different nodes (i.e., network states). One RSN inferred by the pairwise MEM defines an energy landscape. Two nodes are defined to be adjacent by a link if and only if they take the opposite binary activity at just one brain region (i.e., one σ_*i*_; see Figure 1C for the case of *N* = 4).

We analyzed the energy landscape for each RSN using disconnectivity graphs (Becker and Karplus, 1997; Wales, 2010). In short, a disconnectivity graph represents the (dis)connectivity between local minima of the energy. It has also been used to study the Ising spin model, which is equivalent to the pairwise MEM, and its variants (Garstecki et al., 1999; Zhou and Wong, 2009; Zhou, 2011). In the context of the spin systems, a disconnectivity graph with a continuous energy threshold, where the energy threshold is defined in the following, is also referred to as a barrier tree (Fontanari and Stadler, 2002; Hordijk et al., 2003).

We constructed disconnectivity graphs in the following way: (1) A local minimum is a node whose energy is smaller than those of all the *N* neighboring nodes. We exhaustively examined whether each of the 2^*N*^ nodes is a local minimum. (2) We set a threshold energy level, denoted by *E*_th_, to the largest energy level realized by (at least) one of the 2^*N*^ nodes. (3) We removed the nodes whose energy level was higher than *E*_th_. We also removed all links incident to a removed node. In fact, no node or link was removed when the threshold was equal to the largest possible energy level. Some nodes and links were removed when we revisited this step after lowering the *E*_th_ value. (4) We judged whether each pair of local minima was connected by a path in the reduced network. In general, the local minima are classified into some connected components. (5) We repeated steps (3) and (4) after moving *E*_th_ down to the next largest energy level realized by a node. Finally, we obtained a reduced network of the local minima in which each local minimum was isolated. (6) On the basis of these results, we built a disconnectivity graph, i.e., a hierarchical tree whose leaves (i.e., terminal nodes down in the tree) were the local minima. The vertical position of the leaves and internal nodes of the disconnectivity graph represents an energy value. An internal node represents the point at which the branching of different groups of local minima takes place. In other words, local minima that are contained in different branches belong to distinct connected components for an *E*_th_ larger than the value at the common internal root node. Local minima in the different branches belong to the same connected component for *E*_th_ smaller than this value.

### Basin size of local minimum

We then calculated the size of the basin of each local minimum as follows (Stillinger and Weber, 1982, 1984; Becker and Karplus, 1997; Zhou, 2011). We first selected a starting node *i*, which was one of the 2^*N*^ nodes in the network of network states. Then, we identified the neighbor of node *i* possessing the smallest energy level and denoted it by *j*. If *E*(*V*_*j*_) < *E*(*V*_*i*_), we moved to node *j*. This move is in accordance with the steepest descent at node *i*. If such a node *j* did not exist, we remained at node *i*. In the latter case, *i* is a local minimum. If we moved to node *j*, we looked for the steepest descent from node *j* and continued to travel until we arrived at a local minimum. The starting node *i* belongs to the basin of the local minimum that is finally reached. We performed the same procedure for all *i*. The basin size of a local minimum is the fraction of nodes that belong to the basin of the local minimum.

### Energy barrier

For a given disconnectivity graph, we estimated the energy barrier opposing transitions between two local minima denoted by *i* and *j*. Specifically, we defined the energy barrier between *i* and *j* as min [*E*^*b*^ (*V*_*i*_, *V*_*j*_) − *V*_*i*_, *E*^*b*^(*V*_*i*_, *V*_*j*_) − *V*_*j*_], where *E*^*b*^(*V*_*i*_, *V*_*j*_) is the threshold energy level at which the disconnectivity graph branches into a group of nodes that includes *i* and a group that includes *j*. Any path connecting *i* and *j* in the network of network states contains a node whose energy is at least *E*^*b*^(*V*_*i*_, *V*_*j*_). If the energy barrier is high, the transition of network states between *i* and *j* occurs at a small rate at least in one direction. In fact, the transition occurs at different rates in the two directions if *V*_*i*_ and *V*_*j*_ are different (Becker and Karplus, 1997). However, for simplicity, we used the symmetric definition given above (Zhou, 2011).

### Hierarchical clustering

We carried out hierarchical clustering of the brain regions and local minima as follows by using MATLAB. First, we set a distance threshold, *d*_th_ to the smallest Hamming distance realized by a pair of nodes. If the distance between a node pair was equal to or less than the current *d*_th_ value, we bridged the two nodes through a parent node, which is located at *d*_th_ along the axis in the dendrogram. We repeated this procedure by gradually elevating *d*_th_ until all nodes were connected as a single dendrogram.

### Randomized RSNs

As controls, we calculated the disconnectivity graph and other properties of the energy landscape for two types of randomized MEMs. We generated the first type of network by randomly permuting *h*_*i*_ (1 ≤ *i* ≤ *N*) of the original MEM and doing the same for *J*_*ij*_ (= *J*_*ji*_; 1 ≤ *i* ≤ *j* ≤ *N*). We refer to the generated network as a shuffled network. We also generate a second type of randomized network by independently drawing the values of *h*_*i*_ (1 ≤ *i* ≤ *N*) from a normal distribution with the same mean and standard deviation as those of the original MEM and doing the same for *J*_*ij*_ (= *J*_*ji*_; 1 ≤ *i* ≤ *j* ≤ *N*). We refer to the generated network as a Gaussian network.

## Results

### Local minima and the disconnectivity graph

The parameter values of the pairwise MEM inferred for the DMN and FPN are shown in Figure 1A. The distribution of the energy on the basis of all the 2^*N*^ network states is shown in Figure 1B for the two RSNs. The distribution of the energy was unimodal for both RSNs. The shape of the distribution did not significantly differ from that obtained from either of the randomized networks (for both shuffled and Gaussian networks, *P* > 0.6 in the Kolmogorov–Smirnov test; Figure 1B).

**Figure 2:**
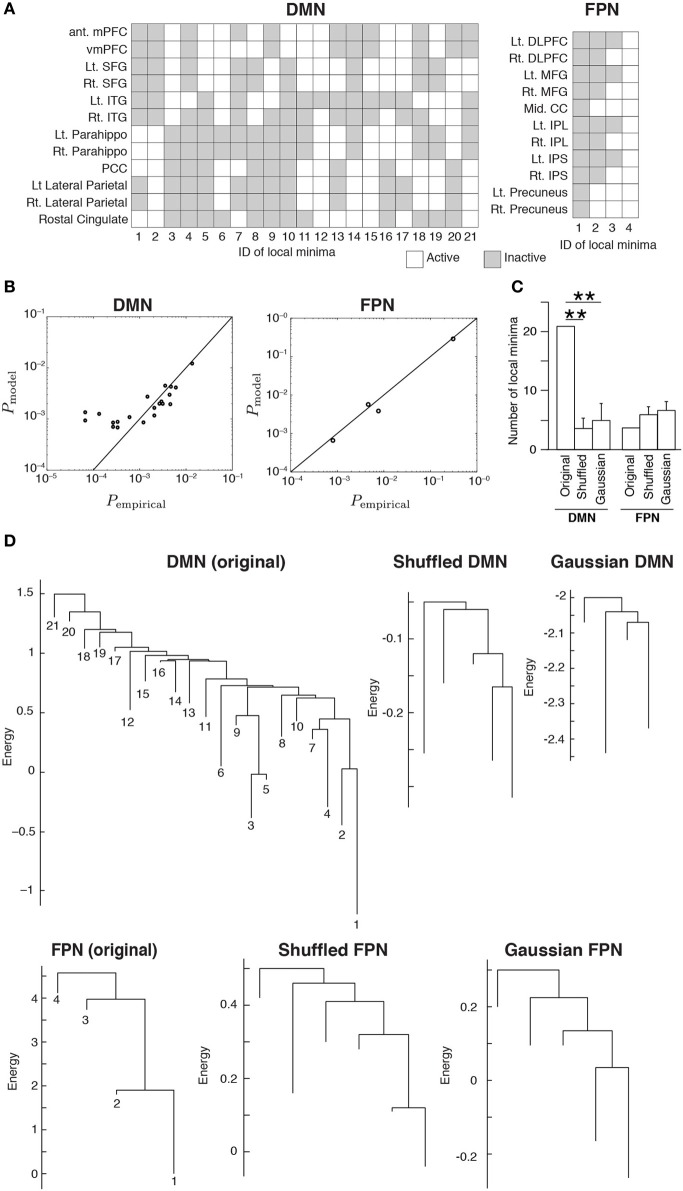
**(A)** Activation patterns of the local minima. The IDs of the local minima are shown on the horizontal axis. The local minima are sorted in order of ascending energy. Each local minimum is specified by an activation pattern, which is an *N*-dimensional binary vector. The white and gray elements indicate active and inactive brain regions, respectively. **(B)** Comparison of the probability that the local minima are realized between the empirical data and the model. Each circle represents a local minimum. **(C)** The number of local minima for the original RSNs and the average number of local minima for the randomized RSNs, where the average is taken over 100 realizations of each type of the randomized networks. Error bars show the standard deviation. ^**^P < 0.01, Bonferroni-corrected. **(D)** Disconnectivity graphs. The vertical axis represents the energy. The numbers immediately under the leaves (i.e., end nodes) represent the IDs of the local minima as defined in panel **(A)**. The energy value at the bottom end of a leaf is equal to that of the corresponding local minimum.

The inferred MEMs for the DMN and FPN had 21 and 4 local minima, respectively. The activity pattern of each local minimum is shown in Figure 2A. In both RSNs, the probabilities that different local minima were visited were similar between the empirical data and the pairwise MEM (Figure 2B). The similarity is particularly evident for the local minima with a low energy (i.e., large probability of the visit) in the DMN. In fact, the error averaged over all 21 local minima in the DMN was 260%. Here, we defined the error for a local minimum as the absolute difference between the empirical and estimated probabilities that the local minimum is realized, divided by the empirical probability. However, the large error was due to three outliers with small probabilities (ID 12, 13, and 16). If the three minima were excluded, the averaged error was 33.2%. Moreover, the error averaged over the 11 local minima with the lowest energy values (i.e., largest probabilities) was 26.2%. In the FPN, the error averaged over all four local minima was 18%. Together with these error values, the results shown in Figure 2B justify the use of local minima of the pairwise MEM in the following analysis as stochastic footprints of the network state.

We calculated the number of local minima for 100 realizations of the two types of randomized RSNs. For the DMN, the number of local minima was significantly larger for the original network than for either type of randomized network (*P* < 0.01, Bonferroni-corrected; Figure 2C). For the FPN, there was no significant difference in the number of local minima between the original and randomized networks.

We then constructed disconnectivity graphs to illustrate relationships between the local minima. The disconnectivity graphs for the original RSN and one realization for each type of randomized network are shown in Figure 2D, separately for the DMN and FPN. In the DMN, the structure of the empirical disconnectivity graph was apparently more complex than that of the randomized networks, partly because the former had more local minima than the latter (Figure 2C). The disconnectivity graph of the DMN has a complex and forked structure relative to that of the FPN. In contrast, the disconnectivity graph of the FPN seems not as complex as the randomized networks and is composed of a single dominant minimum with weak fluctuations, which is one of the main subtypes of the disconnectivity graph (Becker and Karplus, 1997; Wales et al., 1998).

### Clustering of brain regions and local minima

To probe the relationships between different local minima, we performed hierarchical clustering on the basis of similarity between local minima. The (dis)similarity between two local minima was defined by the Hamming distance between the activity patterns of the local minima, i.e., the number of brain regions at which the two local minima possess the opposite binary activity. We constructed a dendrogram for each RSN (see Materials and Methods for the algorithm).

**Figure 3:**
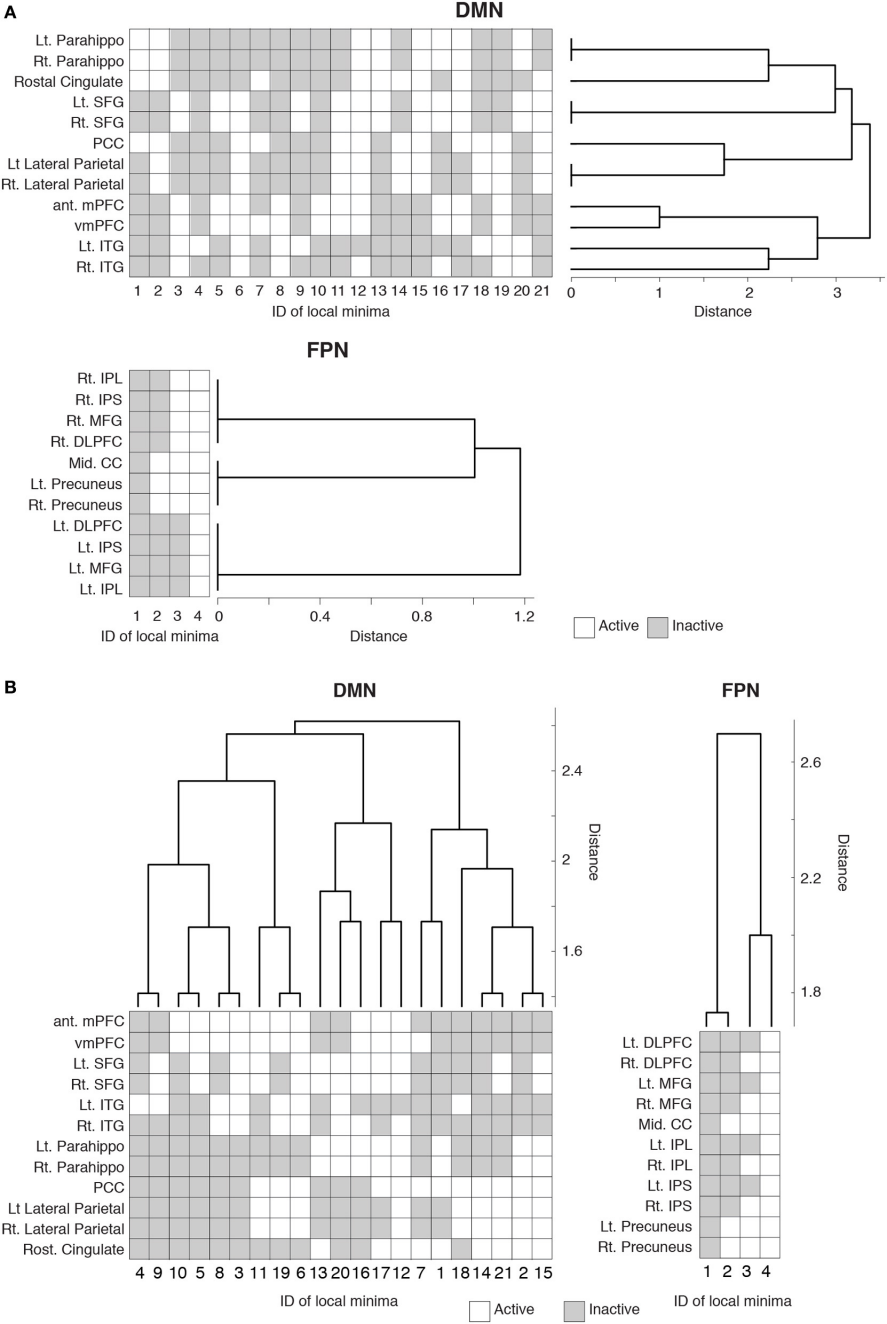
**Hierarchical clustering of the brain regions and local minima.** Each row represents the activity pattern of a brain region in different local minima. Each column represents the activity pattern of a local minimum in different brain regions. **(A)** Dendrogram showing the similarity among the brain regions in a hierarchical fashion. The similarity is measured by the Hamming distance between the activity patterns of two local minima. **(B)** Dendrogram showing the similarity among the local minima.

The dendrogram shown in Figure 3A suggests that, in the DMN, bilateral brain regions show similar activation patterns in most of the local minima. In particular, in the parahippocampal gyri, superior frontal gyri, and lateral parietal region, the bilateral regions had exactly the same activation patterns in all the local minima. In contrast, the resemblance of bilateral regions is uncommon in the FPN. According to the dendrogram, a region in a bilateral region pair was not the nearest to its counterpart, except in the case of the precuneus.

We also quantified the similarity among the local minima by the same hierarchical clustering algorithm (Figure 3B). In the DMN, local minima with the lowest energies (e.g., local min #1 to # 6) were relatively dissimilar. The energy landscape of the DMN is composed of relatively distinct local minima that yield mutlistability. In contrast, in the FPN, the local minima with the lowest energies (e.g., #1 and #2) were more similar to each other than in the case of the DMN. Therefore, we consider that the energy landscape of the FPN is essentially composed of a single global minimum. We provide support of this interpretation in the following sections.

### Size of basin

**Figure 4:**
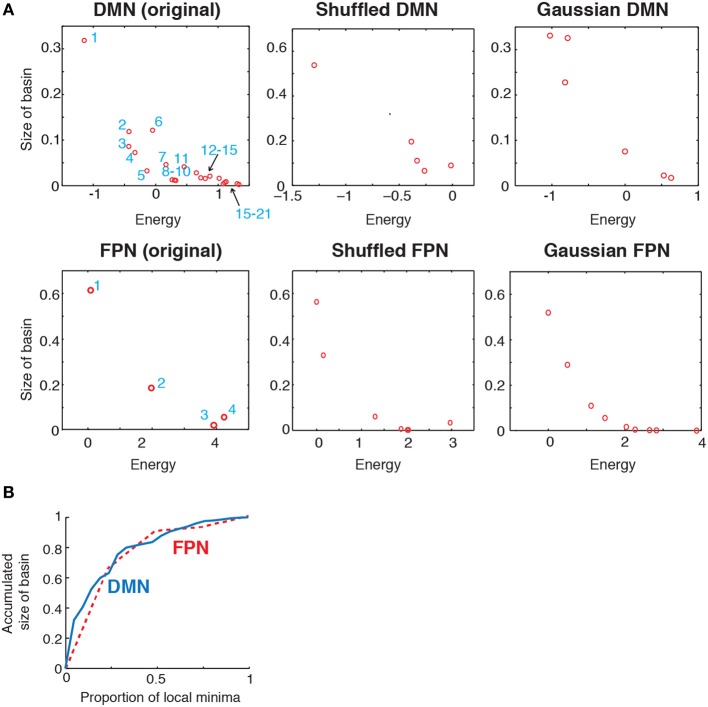
**(A)** Relationship between the size of basin and the energy of local minima. In the two panels on the left, the numbers indicate the IDs of local minima used in Figure 2. **(B)** Accumulated size of the basin for the local minima. The vertical axis shows the fraction of the network states that belong to the basin of one of the local minima with the lowest energies. This quantity is plotted against the fraction of local minima with the lowest energies. The solid and dashed curves indicate the results for the DMN and FPN, respectively.

To further characterize the energy landscape of the two RSNs, we calculated the size of the basin of the local minima. The relationship between the size of the basin and the energy value is shown in Figure 4A. In the figure, an open circle represents a local minimum. In both RSNs, a local minimum with a small energy value tended to have a large basin. This tendency was even stronger in the randomized networks. In both empirical and randomized RSNs, a small number of the local minima with the lowest energy values attracts a majority of the network states (in the sense of the steepest descent walk in the energy landscape). The fraction of network states attracted to one of the local minima with the lowest energies is shown in Figure 4B. For example, when the value at the fraction of local minima is equal to 0.5, the accumulated size of basins is over 0.8; that is, when the half of the local minima with the lowest energies is considered, over 80% of the network states belong to the basin of one of these local minima. In fact, only the six local minima with the lowest energies (ca. 28% of the local minima) attracted more than 80% of the network states in the DMN (solid line in Figure 4B). In the FPN, the local minimum with the lowest energy (25% of the local minima) attracted approximately 60% of the network states (dashed line in Figure 4B).

These results suggest that the lower part of the disconnectivity graph comprising the local minima with the smallest energies, i.e., a connected tree that contains leaves near the bottom in Figure 2D, reflects the backbone of the energy landscape. A visual inspection of Figure 2D reveals that the lower part of the disconnectivity graph for the DMN comprises two main branches, one consisting of local minima labeled 6, 9, 3, and 5, and the other consisting of local minima labeled 8, 10, 7, 4, 2, and 1. In contrast, the lower part of the disconnectivity graph for the FPN is composed of a single main branch.

### Energy barrier

**Figure 5:**
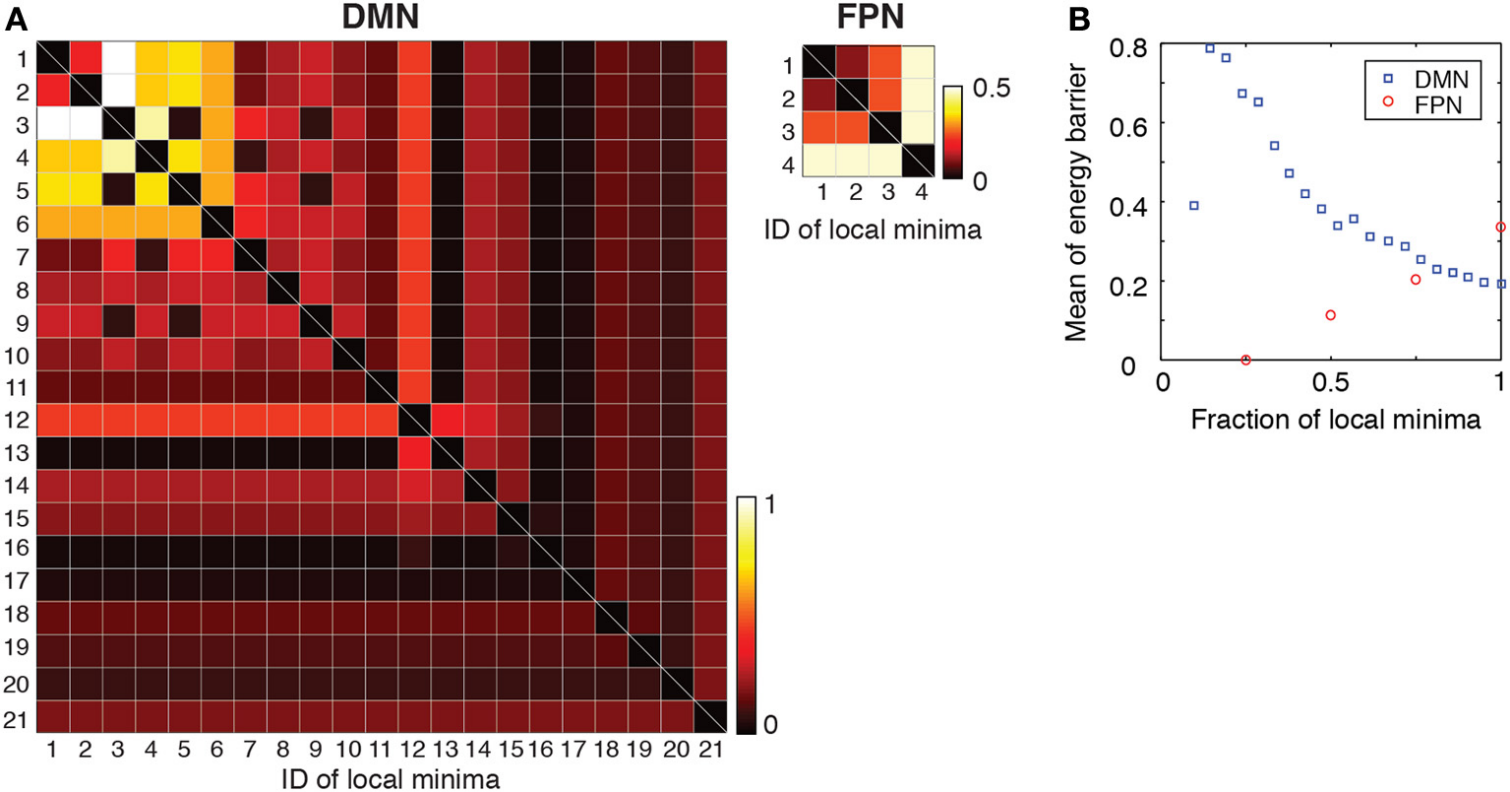
**(A)** Energy barrier between pairs of local minima. The local minima are sorted in order of ascending energy. **(B)** Average of the energy barrier between pairs of local minima with the lowest energies. For example, the values at a fraction 0.5 of local minima indicate the average when we consider only pairs of the local minima whose energies are among the lowest 50%.

To further quantify the difference between the DMN and FPN, we evaluated the transition rates between local minima by calculating the energy barrier between each pair of local minima. If the barrier is high relative to unity, transitions between the two local minima are rare, at least in one direction. The energy barriers for all the pairs of local minima are shown in Figure 5A for each RSN. In the figure, the local minima are sorted according to the energy value. Figure 5A suggests that, in the DMN, transitions among the major local minima accompany high energy barriers such that they occur at small rates. In contrast, in the FPN, transitions between local minima occur relatively easily at least in one direction because of the low barriers that separate them.

Subsequently, we calculated the average of energy barrier between pairs of local minima with the lowest energies. This amounts to averaging the energy barrier values contained in the leading principal minor of the matrix shown in Figure 5A (i.e., top left square submatrix) excluding the diagonal elements. The results are shown in Figure 5B as a function of the size of the minor (i.e., the number of local minima with the lowest energies included in the analysis). As shown in Figure 5B, the averaged energy barrier was much larger in the DMN than in the FPN (*P* < 0.01 in one-sample *t*-tests when the linear size of the minor is 25 and 50% of the entire DMN, respectively. The mean value of the FPN was used as a baseline in the *t*-tests). The difference between the two RSNs was larger when fewer local minima with the lowest energies were considered.

The results shown in Figure 5 imply that, in the DMN, the brain activity may linger in the neighborhood of one of the several local minima for some time and wander from one to another. This interpretation is consistent with the result that the major local minima exhibit distinct activation patterns in the DMN (Figure 3B). In contrast, the brain activity in the FPN may tend to stay near the global minimum albeit with some fluctuations.

## Discussion

We found that the DMN has dominant local minima that are relatively distinct in terms of the activation pattern and an energy barrier of the order of unity separating them from one another. The observed energy barrier is not large enough for the local minima to be justified as metastable states. However, if the brain state gradually changes, it may linger near a major local minimum for some time before transiting to another minimum. Therefore, roughly speaking, the present result is consistent with the concept of the multistable attractor dynamics for the RSN, in which the brain state is considered to travel from one relatively stable state to another, either in a spontaneous manner or triggered by external input (Deco et al., 2012, 2013). Such attractor dynamics may facilitate, for example, large capabilities of computation (Deco et al., 2013). It should be noted that we did not consider dynamics in the present study. Hence, dynamical variants of the present study warrant future work. Accounting for the dynamics will require better temporal resolution in imaging experiments.

In the DMN, the major local minima with small energies and large basins can be roughly classified into two groups separated by a relatively high energy barrier (Figures 3B, 5A). One group consists of the local minima in which the posterior brain regions are activated (local minima #1 and #2) and accounts for approximately 50% of the network states. The other main group consists of those in which the medial prefrontal regions are mainly activated (local minima #3, #4, and #5) and dominates approximately 30% of the network states. Therefore, the DMN is suggested to have two major coarse-grained states marked by posterior-centric activation (the first group) and frontal-centric activation (the second group). Previous studies suggested that the RSNs could be described by attractor dynamics (Deco et al., 2012, 2013). Our empirical evidence indicating the existence of two major coarse-grained states adds to these previous arguments. At a cellular level, multistable neural activity in the hippocampus represents multiple memory items (Leutgeb et al., 2005; Wills et al., 2005; Knierim and Zhang, 2012). The present macroscopic results lend a support to the possibility that multistable activity patterns in the DMN, which includes the parahippocampal cortex, underlie various cognitive functions such as memory maintenance and self referential thought (Raichle et al., 2001; Buckner et al., 2008; Uddin et al., 2009).

In contrast, the energy landscape of the FPN appears to be roughly monostable. In fact, the local minima were separated by low energy barriers (Figure 5A). A possible reason for the absence of multistability is that the activity pattern of the FPN during rest may be different from that when subjects are performing cognitively demanding tasks. The FPN was originally determined as a brain network for attentional cognition (Dosenbach et al., 2006; Corbetta et al., 2008). We should investigate the activity of the FPN during tasks to better understand the FPN.

An obvious limitation of the present study is that we have not directly examined attractor dynamics. Instead, we focused on the energy landscape of the network states constructed from the probability distribution of network states. There are several implicit assumptions underlying our energy landscape analysis. First, the network state was assumed to change gradually. Otherwise, a network state could jump from one local minimum to another by simultaneously flipping the binary states of multiple regions without passing through a network state that realizes the energy barrier. Our analysis, which exploits the concept of the energy barrier, would then be invalidated. The time window for constructing snapshots of brain activity should be small to track possibly step-by-step transitions of the network state. We followed our previous study (Watanabe et al., 2013) and used a time window of approximately 9 s because it was effective at decorrelating different snapshots. It should be noted that the energy landscape does not depend on the temporal resolution if we have sufficiently long data. Analyzing data with improved time resolution may be of interest. We should keep it in mind that the dynamics may not be gradual in fact; non-gradual transition can occur when fMRI signals at different brain regions are tightly synchronized.

Second, an energy barrier analysis implicitly assumes that state transitions depend on the difference between the energy values in the current and subsequent network states. Therefore, we implicitly ignored the effect of past network states on state transitions. To assess the extent of the history dependence of the trajectory is a relevant question. Addressing this question calls for a large amount of data; hence, it should be investigated in tandem with the effect of time window size because correlated snapshots would lead to stronger history dependence under the discrete time frame whose unit is defined by the size of the time window of the measurement.

## Author contributions

Takamitsu Watanabe and Naoki Masuda designed the research. Satoshi Hirose, Hiroyuki Wada, Yoshio Imai, Toru Machida, Ichiro Shirouzu, and Seiki Konishi conducted imaging experiments. Takamitsu Watanabe and Naoki Masuda analyzed the data and wrote the manuscript. Yasushi Miyashita discussed the results and commented on the manuscripts.

### Conflict of interest statement

The authors declare that the research was conducted in the absence of any commercial or financial relationships that could be construed as a potential conflict of interest.

## References

[B1] Allen E. A. Damaraju E. Plis S. M. Erhardt E. B. Eichele T. Calhoun V. D. (2012). Tracking whole-brain connectivity dynamics in the resting state. Cereb. Cortex. [Epub ahead of print]. 10.1093/cercor/bhs352 23146964 PMC3920766

[B2] Barbieri F. Brunel N. (2008). Can attractor network models account for the statistics of firing during persistent activity in prefrontal cortex? Front. Neurosci. 2, 114–122 10.3389/neuro.01.003.2008 18982114 PMC2570072

[B3] Becker O. M. Karplus M. (1997). The topology of multidimensional potential energy surfaces: theory and application to peptide structure and kinetics. J. Chem. Phys. 106, 1495 10.1063/1.473299

[B4] Biswal B. Yetkin F. Z. Haughton V. M. Hyde J. S. (1995). Functional connectivity in the motor cortex of resting human brain using echo-planar MRI. Magn. Reson. Med. 34, 537–541 10.1002/mrm.1910340409 8524021

[B5] Braun J. Mattia M. (2010). Attractors and noise: twin drivers of decisions and multistability. Neuroimage 52, 740–751 10.1016/j.neuroimage.2009.12.126 20083212

[B6] Buckner R. L. Andrews-Hanna J. R. Schacter D. L. (2008). The brain's default network: anatomy, function, and relevance to disease. Ann. N.Y. Acad. Sci. 1124, 1–38 10.1196/annals.1440.011 18400922

[B7] Chang C. Glover G. H. (2010). Time-frequency dynamics of resting-state brain connectivity measured with fMRI. Neuroimage 50, 81–98 10.1016/j.neuroimage.2009.12.011 20006716 PMC2827259

[B8] Corbetta M. Patel G. Shulman G. L. (2008). The reorienting system of the human brain: from environment to theory of mind. Neuron 58, 306–324 10.1016/j.neuron.2008.04.017 18466742 PMC2441869

[B9] Deco G. Jirsa V. Friston K. J. (2012). Principles of Brain Dynamics. Cambridge, MA: MIT Press

[B10] Deco G. Jirsa V. K. McIntosh A. R. (2013). Resting brains never rest: computational insights into potential cognitive architectures. Trends Neurosci. 36, 268–274 10.1016/j.tins.2013.03.001 23561718

[B11] Dosenbach N. U. F. Visscher K. M. Palmer E. D. Miezin F. M. Wenger K. K. Kang H. C. (2006). A core system for the implementation of task sets. Neuron 50, 799–812 10.1016/j.neuron.2006.04.031 16731517 PMC3621133

[B12] Fair D. A. Cohen A. L. Power J. D. Dosenbach N. U. F. Church J. A. Miezin F. M. (2009). Functional brain networks develop from a “local to distributed” organization. PLoS Comput. Biol. 5:e1000381 10.1371/journal.pcbi.1000381 19412534 PMC2671306

[B13] Fontanari J. F. Stadler P. F. (2002). Fractal geometry of spin-glass models. J. Phys. A Math. Gen. 35, 1509 10.1088/0305-4470/35/7/303

[B14] Fox M. D. Snyder A. Z. Vincent J. L. Corbetta M. van Essen D. C. Raichle M. E. (2005). The human brain is intrinsically organized into dynamic, anticorrelated functional networks. Proc. Natl. Acad. Sci. U.S.A. 102, 9673–9678 10.1073/pnas.0504136102 15976020 PMC1157105

[B15] Ganmor E. Segev R. Schneidman E. (2011). Sparse low-order interaction network underlies a highly correlated and learnable neural population code. Proc. Natl. Acad. Sci. U.S.A. 108, 9679–9684 10.1073/pnas.1019641108 21602497 PMC3111274

[B16] Garstecki P. Hoang T. X. Cieplak M. (1999). Energy landscapes, supergraphs, and “folding funnels” in spin systems. Phys. Rev. E Stat. Phys. Plasmas Fluids Relat. Interdiscip. Topics 60, 3219–3226 10.1103/PhysRevE.60.3219 11970130

[B17] Greicius M. D. Krasnow B. Reiss A. L. Menon V. (2003). Functional connectivity in the resting brain: a network analysis of the default mode hypothesis. Proc. Natl. Acad. Sci. U.S.A. 100, 253–258 10.1073/pnas.0135058100 12506194 PMC140943

[B18] Hertz J. Krogh A. Palmer R. G. (1991). Introduction to the Theory of Neural Computation. New York, NY: Addison Wesley Publishing Company

[B19] Honey C. J. Kötter R. Breakspear M. Sporns O. (2007). Network structure of cerebral cortex shapes functional connectivity on multiple time scales. Proc. Natl. Acad. Sci. U.S.A. 104, 10240–10245 10.1073/pnas.0701519104 17548818 PMC1891224

[B20] Hopfield J. J. (1982). Neural networks and physical systems with emergent collective computational abilities. Proc. Natl. Acad. Sci. U.S.A. 79, 2554–2558 10.1073/pnas.79.8.2554 6953413 PMC346238

[B21] Hordijk W. Fontanari J. F. Stadler P. F. (2003). Shapes of tree representations of spin-glass landscapes. J. Phys. A Math. Gen. 36, 3671–3681 10.1088/0305-4470/36/13/302

[B22] Hutchison R. M. Womelsdorf T. Gati J. S. Everling S. Menon R. S. (2013). Resting-state networks show dynamic functional connectivity in awake humans and anesthetized macaques. Hum. Brain Mapp. 34, 2154–2177 10.1002/hbm.22058 22438275 PMC6870538

[B23] Kiviniemi V. Vire T. Remes J. Elseoud A. A. Starck T. Tervonen O. (2011). A sliding time-window ICA reveals spatial variability of the default mode network in time. Brain Connect. 1, 339–347 10.1089/brain.2011.0036 22432423

[B24] Knierim J. J. Zhang K. (2012). Attractor dynamics of spatially correlated neural activity in the limbic system. Annu. Rev. Neurosci. 35, 267–285 10.1146/annurev-neuro-062111-150351 22462545 PMC5613981

[B25] Leutgeb J. K. Leutgeb S. Treves A. Meyer R. Barnes C. A. McNaughton B. L. (2005). Progressive transformation of hippocampal neuronal representations in “morphed” environments. Neuron 48, 345–358 10.1016/j.neuron.2005.09.007 16242413

[B26] Nakagawa T. T. Jirsa V. K. Spiegler A. McIntosh A. R. Deco G. (2013). Bottom up modeling of the connectome: linking structure and function in the resting brain and their changes in aging. Neuroimage 80, 318–329 10.1016/j.neuroimage.2013.04.055 23629050

[B27] Ohiorhenuan I. E. Mechler F. Purpura K. P. Schmid A. M. Hu Q. Victor J. D. (2010). Sparse coding and high-order correlations in fine-scale cortical networks. Nature 466, 617–621 10.1038/nature09178 20601940 PMC2912961

[B28] Rabinovich M. Huerta R. Laurent G. (2008). Neuroscience: transient dynamics for neural processing. Science 321, 48–50 10.1126/science.1155564 18599763

[B29] Rabinovich M. I. Varona P. (2011). Robust transient dynamics and brain functions. Front. Comput. Neurosci. 5:24 10.3389/fncom.2011.00024 21716642 PMC3116137

[B30] Raichle M. E. MacLeod A. M. Snyder A. Z. Powers W. J. Gusnard D. A. Shulman G. L. (2001). A default mode of brain function. Proc. Natl. Acad. Sci. U.S.A. 98, 676–682 10.1073/pnas.98.2.676 11209064 PMC14647

[B31] Santos G. S. Gireesh E. D. Plenz D. Nakahara H. (2010). Hierarchical interaction structure of neural activities in cortical slice cultures. J. Neurosci. 30, 8720–8733 10.1523/JNEUROSCI.6141-09.2010 20592194 PMC3042275

[B32] Schneidman E. Berry M. J. Segev R. Bialek W. (2006). Weak pairwise correlations imply strongly correlated network states in a neural population. Nature 440, 1007–1012 10.1038/nature04701 16625187 PMC1785327

[B33] Shlens J. Field G. D. Gauthier J. L. Grivich M. I. Petrusca D. Sher A. (2006). The structure of multi-neuron firing patterns in primate retina. J. Neurosci. 26, 8254–8266 10.1523/JNEUROSCI.1282-06.2006 16899720 PMC6673811

[B34] Stillinger F. H. Weber T. A. (1982). Hidden structure in liquids. Phys. Rev. A 25, 978–989 10.1103/PhysRevA.25.97821525551

[B35] Stillinger F. H. Weber T. A. (1984). Packing structures and transitions in liquids and solids. Science 225, 983–989 10.1126/science.225.4666.983 17783020

[B36] Tang A. Jackson D. Hobbs J. Chen W. Smith J. L. Patel H. (2008). A maximum entropy model applied to spatial and temporal correlations from cortical networks in vitro. J. Neurosci. 28, 505–518 10.1523/JNEUROSCI.3359-07.2008 18184793 PMC6670549

[B37] Uddin L. Q. Kelly A. M. Biswal B. B. Xavier Castellanos F. Milham M. P. (2009). Functional connectivity of default mode network components: correlation, anticorrelation, and causality. Hum. Brain Mapp. 30, 625–637 10.1002/hbm.20531 18219617 PMC3654104

[B38] Wales D. J. (2010). Energy landscapes: some new horizons. Curr. Opin. Struct. Biol. 20, 3–10 10.1016/j.sbi.2009.12.011 20096562

[B39] Wales D. J. Miller M. A. Walsh T. R. (1998). Archetypal energy landscapes. Nature 394, 758–760 10.1038/29487

[B40] Wang X. J. (2009). Attractor network models. Encycl. Neurosci. 1, 667–679 10.1016/B978-008045046-9.01397-8

[B41] Watanabe T. Hirose S. Wada H. Imai Y. Machida T. Shirouzu I. (2013). A pairwise maximum entropy model accurately describes resting-state human brain networks. Nat. Commun. 4, 1370 10.1038/ncomms2388 23340410 PMC3660654

[B42] Wills T. J. Lever C. Cacucci F. Burgess N. O'Keefe J. (2005). Attractor dynamics in the hippocampal representation of the local environment. Science 308, 873–876 10.1126/science.1108905 15879220 PMC2680068

[B43] Yu S. Huang D. Singer W. Nikolic D. (2008). A small world of neuronal synchrony. Cereb. Cortex 18, 2891–2901 10.1093/cercor/bhn047 18400792 PMC2583154

[B44] Zhou Q. (2011). Random walk over basins of attraction to construct Ising energy landscapes. Phys. Rev. Lett. 106:180602 10.1103/PhysRevLett.106.180602 21635078

[B45] Zhou Q. Wong W. H. (2009). Energy landscape of a spin-glass model: exploration and characterization. Phys. Rev. E 79:051117 10.1103/PhysRevE.79.051117 19518426

